# Supplementary material for: Gut dysbiosis induces the development of mastitis through a reduction in host anti-inflammatory enzyme activity by endotoxemia
Source: Microbiome. 2022 Dec 1;10:205. doi: 10.1186/s40168-022-01402-z (PMC9714159; doi:10.1186/s40168-022-01402-z)
Supplement: Supplementary file 2 — Additional file 1: Figure S1. Serum biochemical characteristics in healthy and mastitis cows. Serum samples from healthy and mastitis cows were collected and performed for biochemical tests. Date were showed as heat map and *p < 0.05 indicates significance by Student’s t test. Figure S2. Mastitis cows have distinct ruminal microbiota structure and functions. A-B. Ace and PD_whole_tree index in the ruminal microbiota showed that cows with mastitis had increased alpha diversity. C. The bacterial composition at the genus level of the ruminal microbiota from healthy and mastitis cows. D. Top 10 bacterial functions of the ruminal microbiota in healthy and mastitis cows. E. PCA score plots for bacterial functions in the ruminal microbiota. F. Tax4Fun analysis shows top 35 bacterial functions in the ruminal microbiota in the indicated groups. Data are expressed as boxplots and **p < 0.01 and ***p < 0.001 indicate significance by Mann-Whitney U test (A-B). Figure S3. Routine blood test and serum biochemical characteristics in RMT mice. A. Routine blood test from control, H-RMT and M-RMT mice showed that M-RMT mice had increased immune activation compared with that of control and H-RMT mice. B. Serum biochemical characteristics showed M-RMT mice had impaired liver function and reduced host ALP. Figure S4. RMT reshapes the gut microbiota structure and functions in recipient mice. A-B. PD_whole_tree and ace index in the gut microbiota showed that the M-RMT group had increased alpha diversity. C. The bacterial compositions at the genus level of the gut microbiota in the indicated mice. D. Top 10 bacterial functions of the gut microbiota in recipient mice. E. PCA score plots for bacterial functions in the gut microbiota. F. Top 35 bacterial functions in the gut microbiota in recipient mice by Tax4Fun analysis. Data are expressed as boxplots and *p < 0.05 indicates significance by Mann-Whitney U test (A-B). Table S1. The oligonucleotides used in this study. [file 40168_2022_1402_MOESM1_ESM.docx]

**Supplementary materials for**

**Gut dysbiosis induces the development of mastitis through a reduction in host anti-inflammatory enzyme activity by endotoxemia**

Caijun Zhao^1#^, Xiaoyu Hu^1#^, Lijuan Bao^1^, Keyi Wu^1^, Yihong Zhao^1^, Kaihe Xiang^1^, Shuang Li^1^, Ying Wang^1^, Min Qiu^1^, Lianjun Feng^1^, Xiangyue Meng^2^, Naisheng Zhang^1^, Yunhe Fu^1^*

1. Department of Clinical Veterinary Medicine, College of Veterinary Medicine, Jilin University, Changchun, Jilin Province 130062, China.

2. Department of Breast Center, West China Hospital, Chengdu, Sichuan University 610041, China.

# Authors contributed equally to the manuscript.

*** Corresponding author:**

Yunhe Fu, E-mail: fuyunhesky@sina.com.


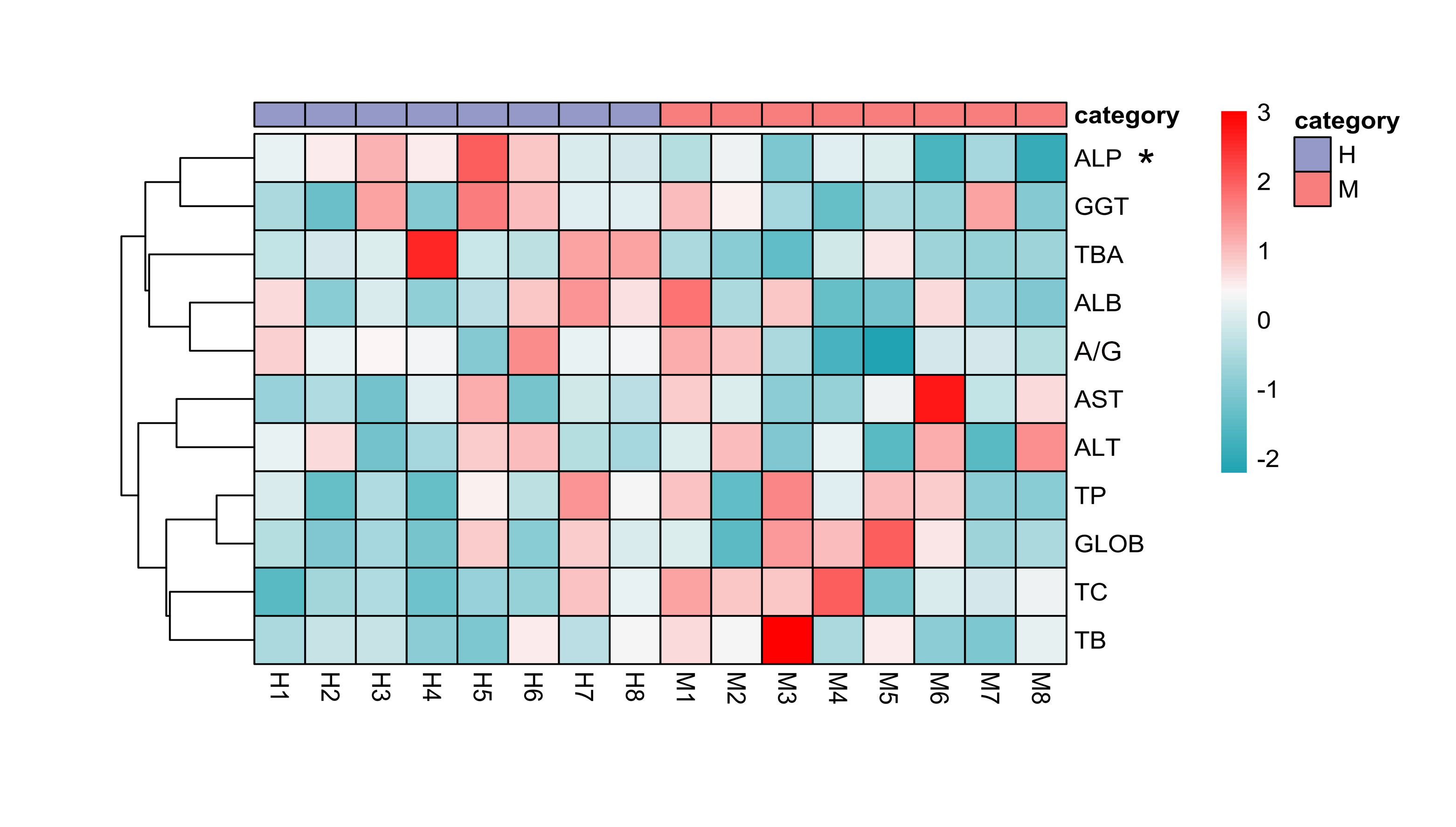


**Supplementary** Fig. **1 Serum biochemical characteristics in healthy and mastitis cows.** Serum samples from healthy and mastitis cows were collected and performed for biochemical tests. Date were showed as heat map and **p* < 0.05 indicates significance by Student’s t test.


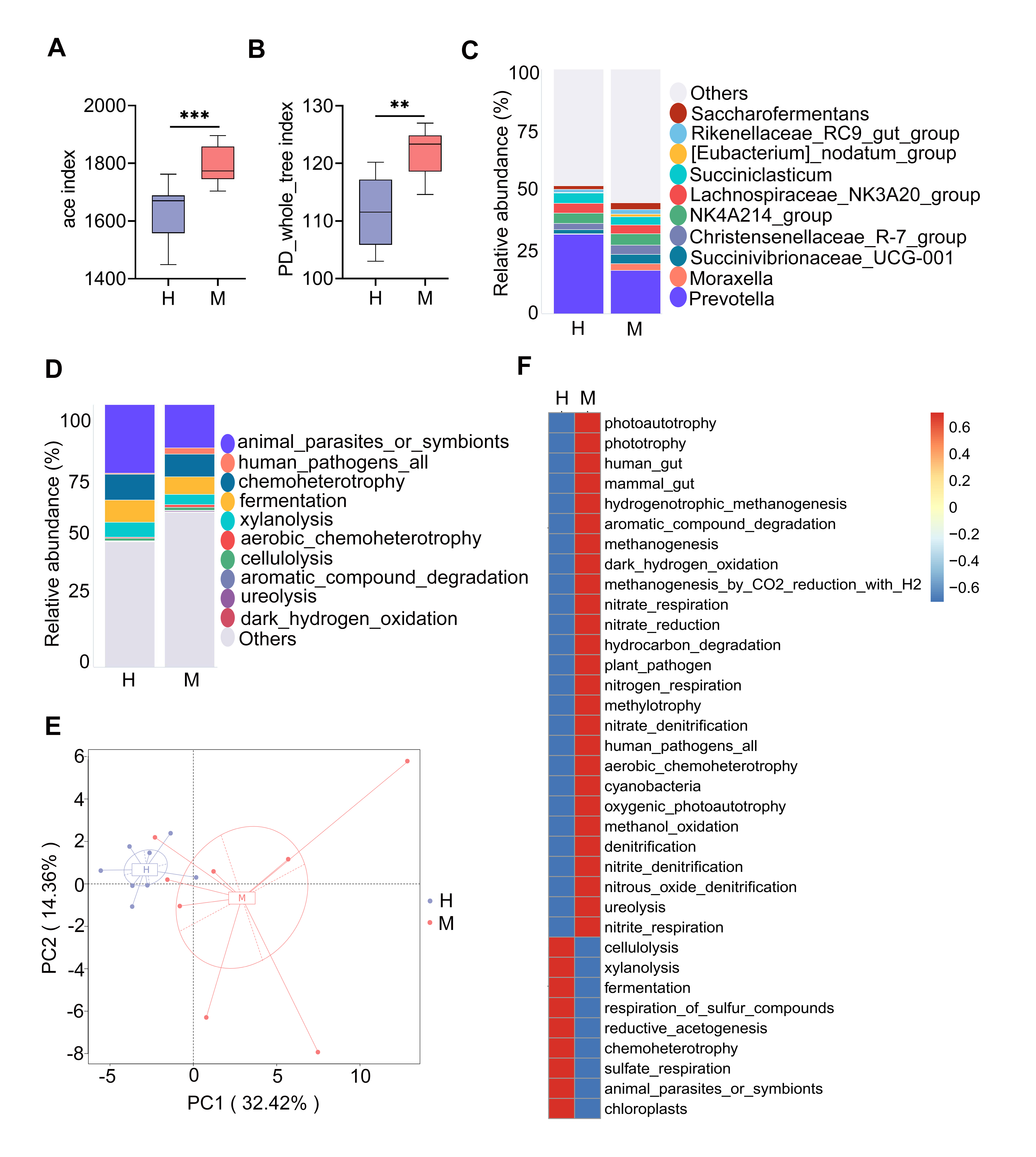


**Supplementary** Fig. 2 **Mastitis cows have distinct ruminal microbiota structure and functions. A-B**. Ace and PD_whole_tree index in the ruminal microbiota showed that cows with mastitis had increased alpha diversity. **C**. The bacterial composition at the genus level of the ruminal microbiota from healthy and mastitis cows. **D**. Top 10 bacterial functions of the ruminal microbiota in healthy and mastitis cows. **E**. PCA score plots for bacterial functions in the ruminal microbiota. **F**. Tax4Fun analysis shows top 35 bacterial functions in the ruminal microbiota in the indicated groups. Data are expressed as boxplots and ***p* < 0.01 and ****p* < 0.001 indicate significance by Mann-Whitney *U* test (**A-B**).


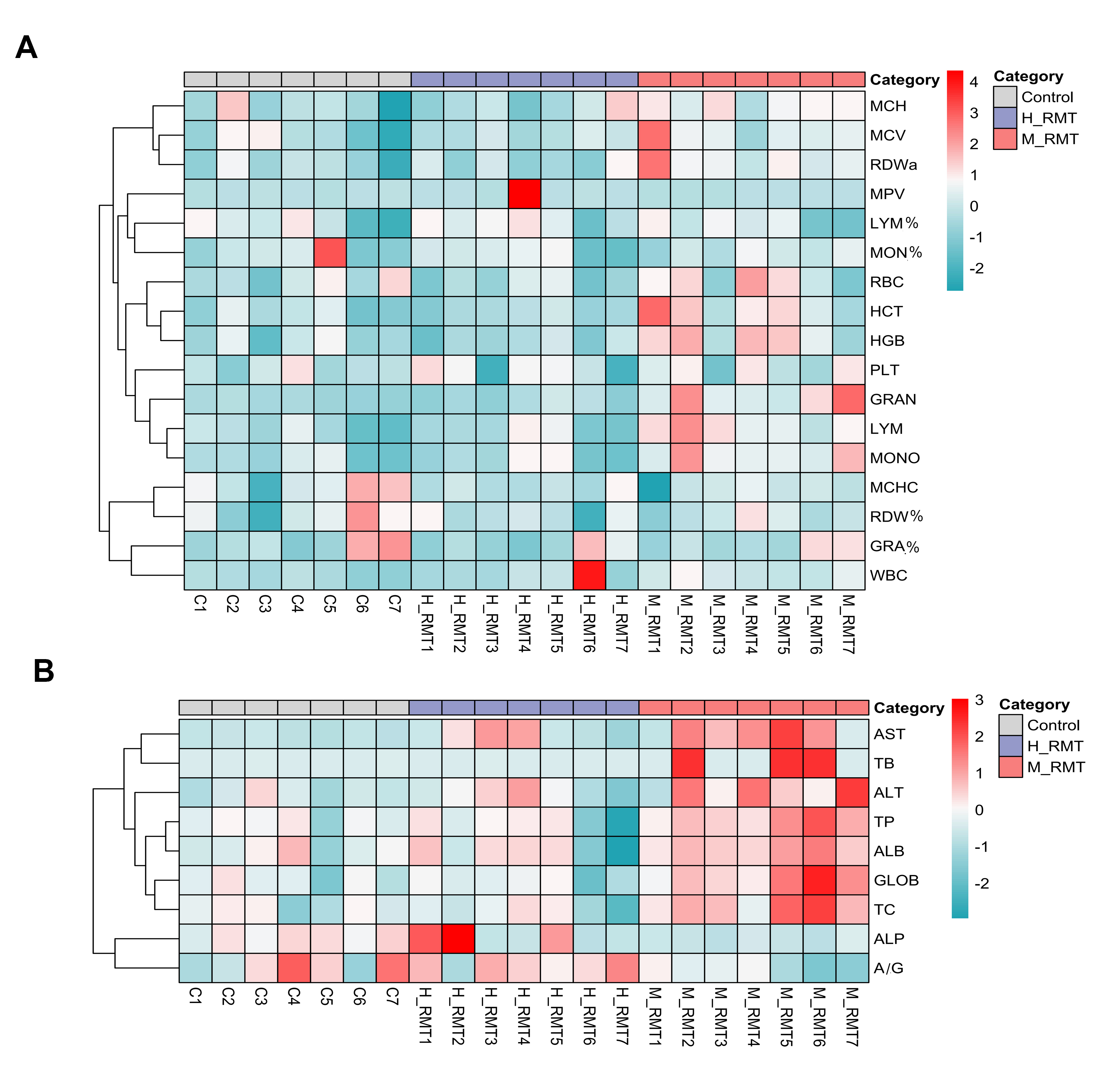


**Supplementary** Fig. **3 Routine blood test and serum biochemical characteristics in RMT mice. A.** Routine blood test from control, H-RMT and M-RMT mice showed that M-RMT mice had increased immune activation compared with that of control and H-RMT mice. **B**. Serum biochemical characteristics showed M-RMT mice had impaired liver function and reduced host ALP.


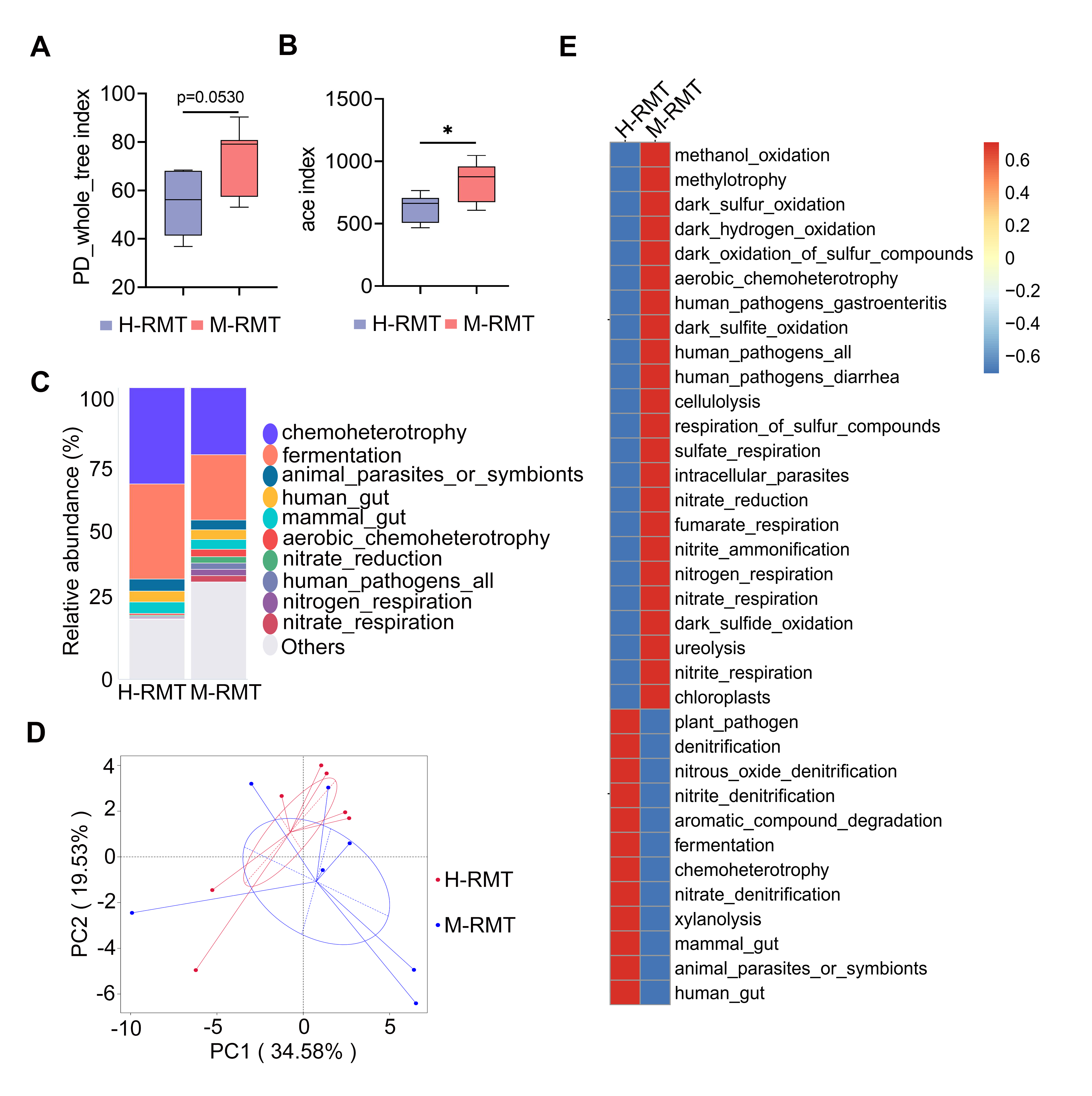


**Supplementary** Fig. **4 RMT reshapes the gut microbiota structure and functions in recipient mice. A-B**. PD_whole_tree and ace index in the gut microbiota showed that the M-RMT group had increased alpha diversity. **C**. The bacterial compositions at the genus level of the gut microbiota in the indicated mice. **D**. Top 10 bacterial functions of the gut microbiota in recipient mice. **E**. PCA score plots for bacterial functions in the gut microbiota. **F**. Top 35 bacterial functions in the gut microbiota in recipient mice by Tax4Fun analysis. Data are expressed as boxplots and **p* < 0.05 indicates significance by Mann-Whitney *U* test (**A-B**).

**Supplementary Table 1. The oligonucleotides used in this study.**

| Gene | Primer | Sequence(5′ to 3′) |
| --- | --- | --- |
| *TNF-α* | sense | 5′- CCCTCACACTCAGATCATCTTCT-3′ |
|  | antisense | 5′- GCTACGACGTGGGCTACAG-3′ |
| *IL-1β* | sense | 5′- GCAACTGTTCCTGAACTCAACT-3′ |
|  | antisense | 5′-ATCTTTTGGGGTCCGTCAACT-3′ |
| *IL-6* | sense | 5′- TAGTCCTTCCTACCCCAATTTCC-3′ |
|  | antisense | 5′-TTGGTCCTTAGCCACTCCTTC-3′ |
| *CCL2* | sense | 5′-TTAAAAACCTGGATCGGAACCAA-3′ |
|  | antisense | 5′-GCATTAGCTTCAGATTTACGGGT-3′ |
| *Tjp-1* | sense | 5′-GCCGCTAAGAGCACAGCAA-3′ |
|  | antisense | 5′-TCCCCACTCTGAAAATGAGGA-3 |
| *Occludin* | sense | 5′-CCCAGGCTTCTGGATCTATGT-3′ |
|  | antisense | 5′-TCCATCTTTCTTCGGGTTTTCA-3 |
| *Claudin-3* | sense | 5′-ACCAACTGCGTACAAGACGAG-3′ |
|  | antisense | 5′-CAGAGCCGCCAACAGGAAA-3 |
| *GAPDH* | sense | 5′-AACTTTGGCATTGTGGAAGG-3′ |
|  | antisense | 5′-ACACATTGGGGGTAGGAACA-3′ |
